# Predicting Spray Dried Dispersion Particle Size Via Machine Learning Regression Methods

**DOI:** 10.1007/s11095-022-03370-3

**Published:** 2022-08-19

**Authors:** John M. Schmitt, John M. Baumann, Michael M. Morgen

**Affiliations:** 1 Computational Science, Lonza, 1201 NW Wall St, Bend, OR 97703 USA; 2 Global Research and Development, Lonza, Bend, OR USA

**Keywords:** amorphous solid dispersion, machine learning, particle size, spray drying

## Abstract

Spray dried dispersion particle size is a critical quality attribute that impacts bioavailability and manufacturability of the spray drying process and final dosage form. Substantial experimentation has been required to relate formulation and process parameters to particle size with the results limited to a single active pharmaceutical ingredient (API). This is the first study that demonstrates prediction of particle size independent of API for a wide range of formulation and process parameters at pilot and commercial scale. Additionally we developed a strategy with formulation and target particle size as inputs to define a set of “first to try” process parameters. An ensemble machine learning model was created to predict dried particle size across pilot and production scale spray dryers, with prediction errors between −7.7% and 18.6% (25th/75th percentiles) for a hold-out evaluation set. Shapley additive explanations identified how changes in formulation and process parameters drove variations in model predictions of dried particle size and were found to be consistent with mechanistic understanding of the particle formation process. Additionally, an optimization strategy used the predictive model to determine initial estimates for process parameter values that best achieve a target particle size for a provided formulation. The optimization strategy was employed to estimate process parameters in the hold-out evaluation set and to illustrate selection of process parameters during scale-up. The results of this study illustrate how trained regression models can reduce the experimental effort required to create an in-silico design space for new molecules during early-stage process development and subsequent scale-up.

**Supplementary Information:**

The online version contains supplementary material available at 10.1007/s11095-022-03370-3.

## Introduction

Application of model-based approaches during pharmaceutical drug development has been successful at reducing time, cost and raw materials required to obtain the required product quality [1, 2]. Additionally, this approach is synergistic with regulatory guidance including the quality by design (QbD) paradigm specified by ICH Q8 and the more recent Pharma 4.0™ developed by ISPE [3, 4].

Machine learning models have recently been evaluated for use in pharmaceutical development across a broad range of areas, including drug discovery and drug formulation and to a lesser extent manufacturing processes [5–9]. These approaches have demonstrated the utility of these techniques to reduce experimental burden and codify prior knowledge by using a broad range of routinely collected data.

Spray drying is employed in the pharmaceutical industry as a single continuous unit operation in which a solution containing dissolved active pharmaceutical ingredient (API) and excipients is atomized into a drying chamber in conjunction with a high temperature gas, resulting in a dried powder. For oral drug delivery, spray drying is often used to address formulation challenges associated with bioavailability-limited drugs, such as slow dissolution rate or low solubility in the gastrointestinal tract. In such cases, spray drying improves bioavailability by creating a high energy amorphous spray dried solid dispersion (SDD), where the excipients stabilize the API in an amorphous state within the excipient matrix [10]. Further details regarding the spray drying equipment, process, components and parameters are available in the literature [11–16].

SDD particle size is considered a critical quality attribute that can impact performance and manufacturability. Smaller particle sizes have a larger surface area that increases dissolution rate, which can improve bioavailability [17]. However, smaller particle sizes can cause manufacturing challenges through reduced particle compressibility and poor flow properties, increasing the risk of particle segregation and compressibility adversely affecting final dosage form qualities, such as content uniformity and tablet hardness [18, 19]. Substantial experimentation is required to co-optimize formulation and process parameters to define the multivariate relationships for a SDD formulation [20, 21]. Design of experiment (DOE) approaches are typically employed; however, the resulting model is specific to a single API, formulation or process scale [20].

There is limited prior research to predict or model spray dried particle size for ASDs. In a review of scale-up for pharmaceutical spray drying, Poozesh and Bilgili evaluated published studies and found the majority of these studies have been conducted at laboratory scale with two-fluid nozzles. This presents a challenge translating to larger scale spray dryers using pressure nozzles and also limits the particle size achievable due to the small chamber and short residence time [22]. Particle size in these studies with two fluid-nozzles are typically less than 10 μm, *versus* particle size data from SDDs made with pressure nozzles which can range from 10 to 100 μm. The most relevant examples evaluated the following approaches: 1) statistical models from a traditional DOE to identify impact of process parameters and formulation attributes on particle size; 2) fundamental or empirical models to predict droplet size and subsequently relate that to particle size; and 3) experimental approaches to measure droplet size and relate that to particle size. In the first, a case study is presented around SDD manufacture with pressure swirl nozzles detailing screening, optimization and robustness studies [23]. During screening and subsequent optimization studies, it was found that a linear model did not adequately describe the particle size results, however, in the optimization stage a good correlation was observed by including quadratic terms using outlet temperature and feed pressure as variables. This model was successful for a given formulation and process scale but would not be expected to translate well to new formulations since there are no parameters that describe these variables. Another statistical model from a DOE was developed to map the process space and predict particle size of a ProCepT Micro laboratory scale spray dryer using a dispersion polymer placebo solution consisting of HPMCAS-LF in acetone [24]. This model was found to not generalize well when applied to SDDs with other polymers or when API was used in the formulation. In the second example, a mechanistic model was proposed by applying hydrodynamic instability theory that was fit using published droplet size data collected with the same two-fluid nozzle geometry using a laboratory scale spray dryer [25]. Due to the empirical fit of the fundamental model with data specific to the nozzle used in the study, it would not be expected to extrapolate outside of the formulations and nozzle used in the study. Empirical models to predict droplet size are shown for SDD manufacture using pressure nozzles and can be used with good results, but extrapolation beyond the 12 nozzle geometries used to develop the correlation may be limited [23]. However, the empirical droplet size model does contain formulation attributes (e.g. spray solution viscosity) that could aid in some extrapolation. In the final example, an SDD scale-up case study measured droplet size by laser diffraction (Malvern Spraytec) to develop an empirical droplet size model intended to match particle size between a pilot and production scale spray dryer both equipped with two-fluid nozzles [26]. Atomization conditions were selected to produce equivalent droplet sizes, but it was found that particle size increased from 50% to 100% between the two scales using this approach and additional experimentation at production scale was required to match particle size.

In this study machine learning models are created to directly predict the median SDD particle size from spray solution and process parameters, across a diverse set of APIs and formulations. These models were applied to both PSD-1 (pilot scale - nominal drying gas flowrate 100 kg/hr) and PSD-2 (production scale - nominal drying gas flowrate 450 kg/hr) scale dryers (GEA Process Engineering A/S) specific to pressure swirl atomization. To the best of the authors’ knowledge, this represents the first work to predict dried particle size across a combination of APIs, polymers, solvents, nozzles, dryer scales and process conditions. Although the models are derived empirically, the functional relationships between formulation parameters, process parameters and particle size can be understood using Shapley additive explanations, and are consistent with mechanistic understanding and prior research of droplet and particle formation and its relation to particle size. Separately, an optimization strategy is demonstrated where the desired particle size and formulation parameters are provided as inputs into the model and used to predict “first to try” process parameters to best achieve the target particle size.

## Materials and Methods

### Data Compilation

Regression model development was performed in a retrospective fashion using historical process and formulation data recorded for 680 SDD lots using 57 different pressure nozzles across two spray dryer scales (505 lots on PSD-1, 175 lots on PSD-2) and 88 unique APIs. Particle size distributions for each lot were measured with either a Malvern Mastersizer 2000 or Mastersizer 3000 (Malvern Instrument Ltd., Worcestershire, UK), where volumetric median size (D50) was recorded as the mean of three measurements. Dry dispersion methods were employed for these measurements, with a range of parameters selected to result in a fit-for-purpose method that was formulation-dependent. Formulation and process parameters recorded for each experiment are provided in Table I.

**Table I:** Spray Drying Formulation and Process Attributes Available for Model Development

	Attributes	Units	Ranges/values present in data
Formulation	Active loading (dry basis)	Weight percent	5.0–100.0
	Polymer loading in solution	Weight percent	0.0–19.5
	Spray solution solids content	Weight percent	0.6–30.0
	Solvent(s)	Weight percent	0.0–100.0
	Polymer type	None	HPMC E3, HPMCAS H*, HPMCAS L*, HPMCAS M*, HPMC-P, PVP VA64, Eudragit L100, CAP, None
	Solvent type	None	Acetone, DCM, Methanol, THF, Water
Process parameters	Spray dryer inlet temperature	Degrees Celsius	52.5–180.0
	Spray dryer outlet temperature	Degrees Celsius	9.0–66.8
	Drying gas flow rate	g/min	500.0–8500.0
	Spray solution to drying gas flow rate ratio	None	0.019–0.27
	Atomization pressure	MPa	0.14–7.03
	Pressure Nozzle employed	None	57 unique nozzles (by name)
	Spray dryer scale	None	PSD-1, PSD-2
Output	Dried particle size	Microns	8.0–104.0

### Regression Model Development

**Fig. 1:**
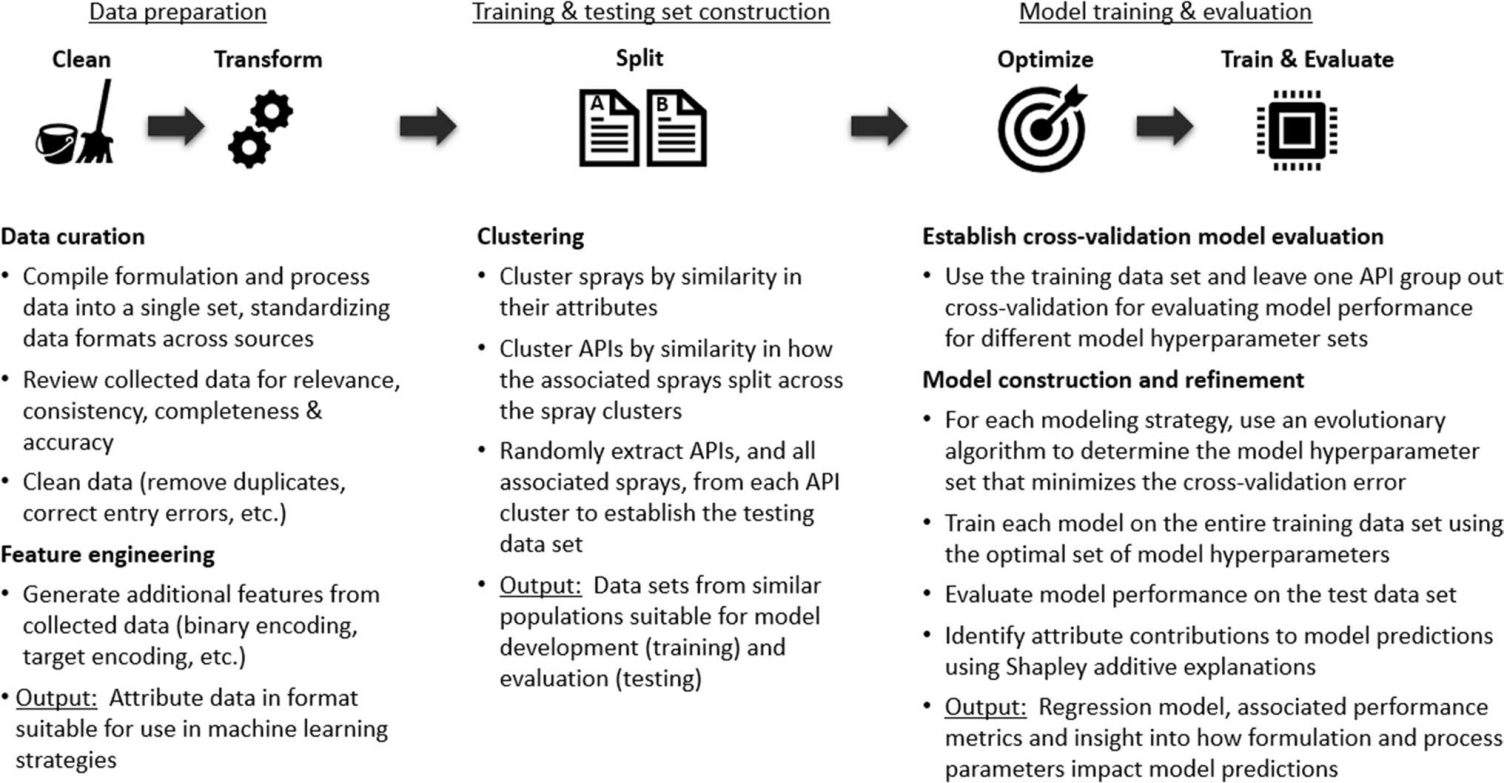
Overview of the particle size regression model development workflow.

The aggregated formulation and process data were used to develop a regression model to predict median dried particle size. The model development workflow is summarized in Fig. 1 and detailed in the following subsections.

#### Data Preparation

For model development, non-numeric data had to be converted to a numeric format. Categorical variables, such as polymer and excipient identities, were binary encoded to aid in splitting data into training and testing data sets. Binary encoding created a new column for each unique polymer and solvent. If a specific solvent or polymer was employed in a spray, then a value of one was entered in the associated column, otherwise the value was set to zero. As more fidelity could be extracted for the particle size prediction model through weight percent values, these binary values were converted to continuous values for regression model development by multiplying the associated weight percent by the binary encodings.

Binary encoding was not used for spray nozzles to avoid model overfitting, as 57 unique nozzles were used across sprays. However, as nozzle selection was believed to impact dried particle size, a target encoding with additive smoothing was used to generate a single numeric nozzle column for regression modeling [27]. The intention of employing target encoding was to capture gross trends in particle size changes with nozzle type, reflecting relationships between internal nozzle geometry and fluid flow or fluid properties that were not captured by other attributes but have been reported previously [28].

The primary concern with employing target encoding is data leakage leading to model overfitting, which would be characterized by poor model performance on previously unseen data (i.e. the test set). Both additive smoothing and leave-one-group-out cross-validation were employed in the target encoding process to prevent data leakage that would lead to model overfitting. As detailed in the Supplemental Material, the use of additive smoothing with target encoding replaced the categorical nozzle identifier with a numeric value that was the weighted average of: a) the mean particle size for all sprays conducted with the nozzle and b) the mean particle size for all sprays (across all nozzles, i.e. the overall mean particle size) in the data set. As the number of sprays associated with a nozzle increased, the encoded value depended more on the average particle size achieved with that nozzle and less on the overall mean particle size. However, the probability of overfitting the training data set was reduced by the fact that an increase in sprays was typically associated with a wider expected range of particle sizes for the nozzle, which was driven by an increase in the variety of APIs, formulation and process parameters. To further prevent data leakage, the target encoding was established only from data present in each intermediate training data set in cross-validation (CV) and on the training data set in final model training. In each case, the target encoding was determined only from the associated training data. The result was a lookup table that mapped a nozzle identifier to an encoded value, which was subsequently used to determine the encoded value for each instance in the test data. In this fashion, no information about the target in the test data set was employed in the associated model prediction. While target encoding is an advanced machine learning technique due to the extra effort required to prevent overfitting, it has been incorporated in various forms in machine learning platforms, packages and modeling strategies [29–31]. However, to illustrate the benefit of employing target encoding, while also alleviating concerns that model performance was achieved solely due to target encoding, models were also constructed without target encoding but with the addition of the nozzle orifice diameter as an input attribute.

To ensure all attributes had similar scales during model development, minmax scaling was used to normalize each attribute of the training data set to within a range between 0 and 1. The identified scaling relationships were subsequently used to transform each attribute of the test data set in a similar fashion.

#### Training and Testing Set Construction

The aggregated data was split into training and testing data sets, with model development and evaluation performed on the training and testing data sets, respectively. As API interactions with polymers and solvents can change properties that impact droplet and particle size, all sprays for a given API were placed in either the training set or testing set, but not split across both [12]. Splitting by API was done to reduce the chance of data leakage, in which patterns specific to API/excipient interactions and particle size in the test set are known in the training set. Data leakage of this sort can lead to an overly optimistic estimation of model performance that might not hold for new APIs. For new APIs, the model will have no advance knowledge of formulation and API interactions that could affect particle size, so it is important to train the predictive model in a similar fashion.

Clustering was employed in data splitting to better ensure that similar populations of APIs/sprays existed in the training and testing sets, such that differences between training and testing performance were more likely to be due to model overfitting than differences in populations between the data sets. The clustering strategy, detailed in the Supplemental Material, yielded API clusters that were used to establish the data sets for model development and evaluation. The testing data set was established by extracting 25% of the APIs, and associated sprays, from each of these final clusters. The training data set was comprised of the APIs, and associated sprays, remaining after this extraction.

#### Model Training and Evaluation

**Fig. 2:**
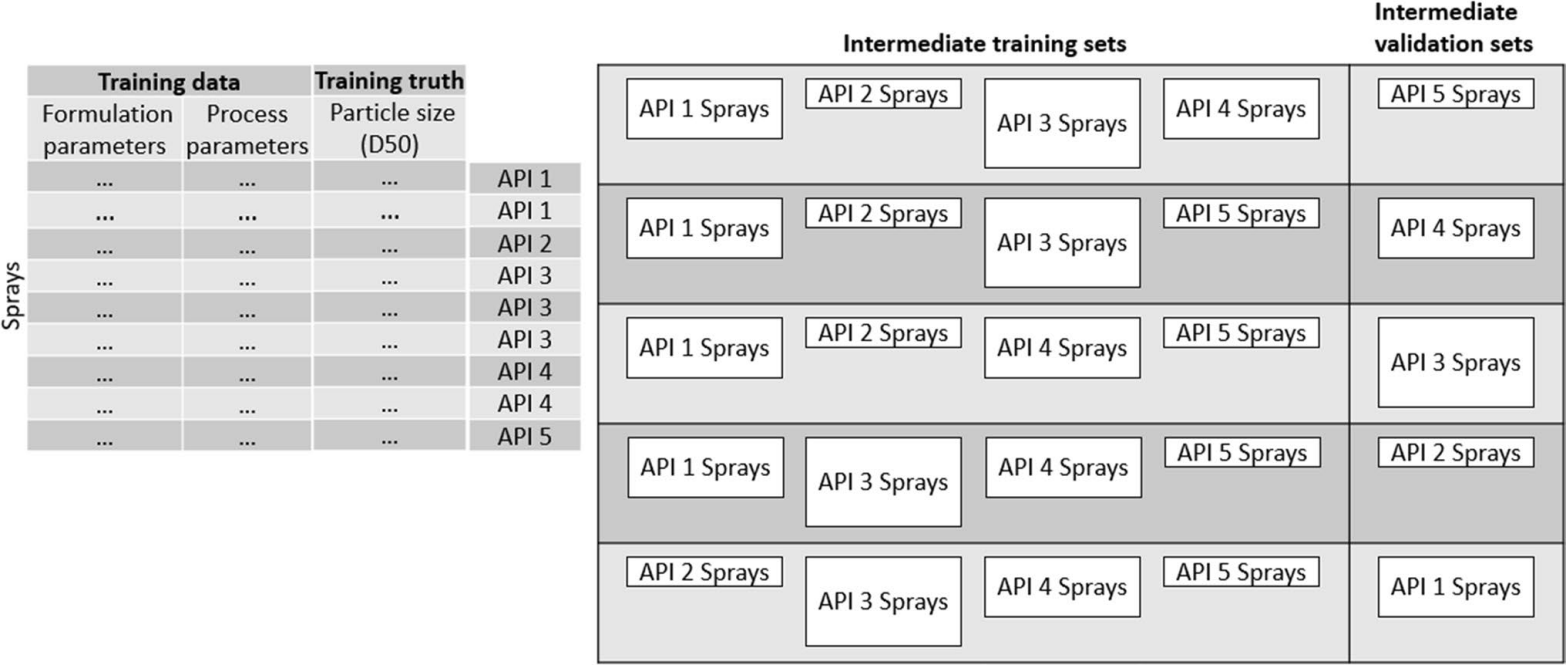
Illustrative example of leave-one-API-out cross-validation in the case of a training data set composed of sprays from five APIs. Intermediate training data sets are constructed by removing all of the sprays associated with a particular API to an intermediate validation data set. A model is constructed on each intermediate training data set and evaluated on the associated intermediate validation data set. Overall cross-validation performance is determined as the average performance on the intermediate validation data sets.

Machine learning model development requires identification of the model hyperparameter values that yield the best performance without overfitting the patterns present in the training data set. Model hyperparameters are externally prescribed values, specific to a machine learning algorithm, that impact how the model is trained or the capacity of the model to learn patterns in the data. Changes in these hyperparameters can yield dramatic differences in model performance, which necessitates use of a cross-validation strategy to aid in preventing model overfitting. To reduce the possibility of overfitting the patterns present in the training data set, leave-one-group-out cross-validation was employed on the training data set during model hyperparameter optimization (Fig. 2). The leave-one-group-out strategy treated all sprays associated with a particular API in the training data set as a group during cross-validation. For a particular selection of model hyperparameters, sprays associated with a single API were extracted as an intermediate validation set and a model was built from the data associated with all remaining sprays. This process was repeated with sprays from each unique API serving as the intermediate validation set in turn. Overall cross-validation performance for a particular set of model hyperparameters was determined as the average model performance across each validation set. Model hyperparameter optimization was performed using an evolutionary algorithm to optimize the leave-one-group-out cross-validation performance on the training data set, as detailed in the Supplemental Material. Linear and nonlinear regression methods were trained to predict dried particle size using the following strategies in the Python programming language: linear regression, lasso, ridge, elastic net, partial least squares, multivariate adaptive regression splines, support vector regression, kernel ridge regression, random forest, extreme gradient boosted trees and neural networks.

#### Model Interpretation

The adoption of machine learning models in the pharmaceutical industry has often been hampered by incomplete or limited data sets and model overfitting. Related, it can be difficult to explain to non-experts how complex machine learning models arrive at their prediction from the provided input values. In this study, model overfitting has been addressed through the adoption of appropriate cross-validation techniques for identifying model hyperparameters and clustering for splitting provided data into training and testing data sets with similar populations. While linear models enable easy interpretation via inspection of attribute coefficients, it is more difficult to identify how individual attributes contribute to the overall prediction for more complex models that may exhibit significant improvements in predictive performance, such as neural networks, support vector machines or random forests. Linear models also require design of experiments approaches leading to a large experimental burden for evaluating parameter ranges that may or may not be applicable to the final design space. Machine learning has a great potential to develop these models from large data sets of routinely collected formulation and process attributes from pharmaceutical processes.

In this work, Shapley additive explanations (SHAP) were employed for model interpretability. Specifically, SHAP values were used to deconstruct overall model predictions of particle size into individual input attribute contributions (e.g. formulation parameters, process parameters, etc.) [32]. The SHAP approach enforces local accuracy and consistency properties, such that SHAP value estimates of attribute contribution to the prediction must sum to the overall model prediction. Because of their additive nature, SHAP values can be employed to obtain insight into attribute importance at both a local and global level. At a local level, SHAP values provide information as to the marginal contribution of each attribute to the model prediction for a given instance, thereby providing insight into how an individual model prediction was obtained from the input attributes. At a global level, aggregation of the local SHAP values over all dataset instances provide insight into global attribute variation patterns and their impact on model predictions. SHAP values easily identify important relationships that lead to deeper understanding of the connection between model attributes and model prediction. In many cases, these correlations could be identified *a priori* by a subject matter expert, however, by looking at the model contributions in this fashion, less obvious attributes can be extracted out of the model to provide a framework for subsequent mechanistic understanding or optimization studies.

### Model Inversion

The particle size prediction model can be leveraged during process development to iterate on a set of process parameters to achieve a target particle size. An application of the model is to identify acceptable ‘first to try’ process parameters to achieve a specified particle size for a given formulation. Using the model in this fashion could reduce the experimentation required to achieve a desired particle size, especially when combined with the process knowledge of subject matter experts. This also aligns with the QbD paradigm enabling initial construction of a virtual design space that is later confirmed and optimized through reduced experimentation.

A two phase strategy was employed to ‘invert’ the model and identify initial experimental process parameters that would lead to a target particle size based on defined formulation attributes. In the first phase, in the absence of any provided constraints on the process parameters by scientists, lower and upper bounds on each process parameter were established by a multiple iterative imputation strategy [33, 34]. This iterative imputation strategy creates a series of regression models to predict the unknown process parameters for a spray using the training data set. These models are created ‘on demand’ for each new spray and are unrelated to the developed particle size prediction model. In the second phase, the process parameters were optimized within these bounds to produce a model-based particle size prediction close to the target particle size while also adhering to formulation/process parameter patterns present in historical data.

**Fig. 3:**
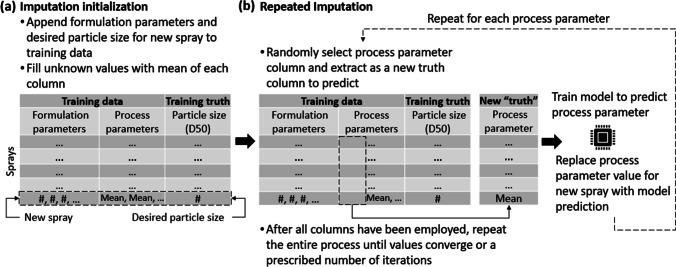
Overview of repeated imputation strategy that determines initial process parameter values that define the search space for the model inversion optimization. (**a**) The imputation is initialized via specification of the formulation parameters and desired particle size, with initial values for the process parameters determined as the mean of the values obtained from the training data set. (**b**) In repeated imputation, each process parameter column is extracted in turn as a new output column to be predicted. In each case, a model is trained to predict the process parameter column and the associated prediction replaces the prior process parameter value for the new spray. This process is repeated in its entirety until the process values for the new spray converge or a specified number of iterations have elapsed.

The first phase (Fig. 3) treats the determination of process parameters as a missing data problem where the formulation parameters and target particle size are known for the spray but the process parameters are missing. This initial phase could be removed entirely and replaced by scientist-provided constraints on each process parameter, which would likely improve inversion results. In this study, a worst case scenario was investigated in which such constraints were assumed to be unknown and this process was employed instead to identify a smaller search space for the subsequent optimization. After appending the known spray information onto the training data set, the multiple iterative imputation strategy creates a regression model of each process parameter in turn as a function of the other features. Each missing process parameter for the spray is initially replaced with the mean value of those present in the training set and imputed sequentially in a randomized order, where previously imputed process parameter values are subsequently employed in modeling other process parameters. This process is repeated to improve the estimates for each of the missing process parameters for a specified number of iterations, or a convergence threshold, whichever is reached first. Upon completion, the realized process parameters are employed in conjunction with prescribed percentage differences to establish minimum and maximum bounds on each process parameter for the subsequent optimization stage.

**Fig. 4:**
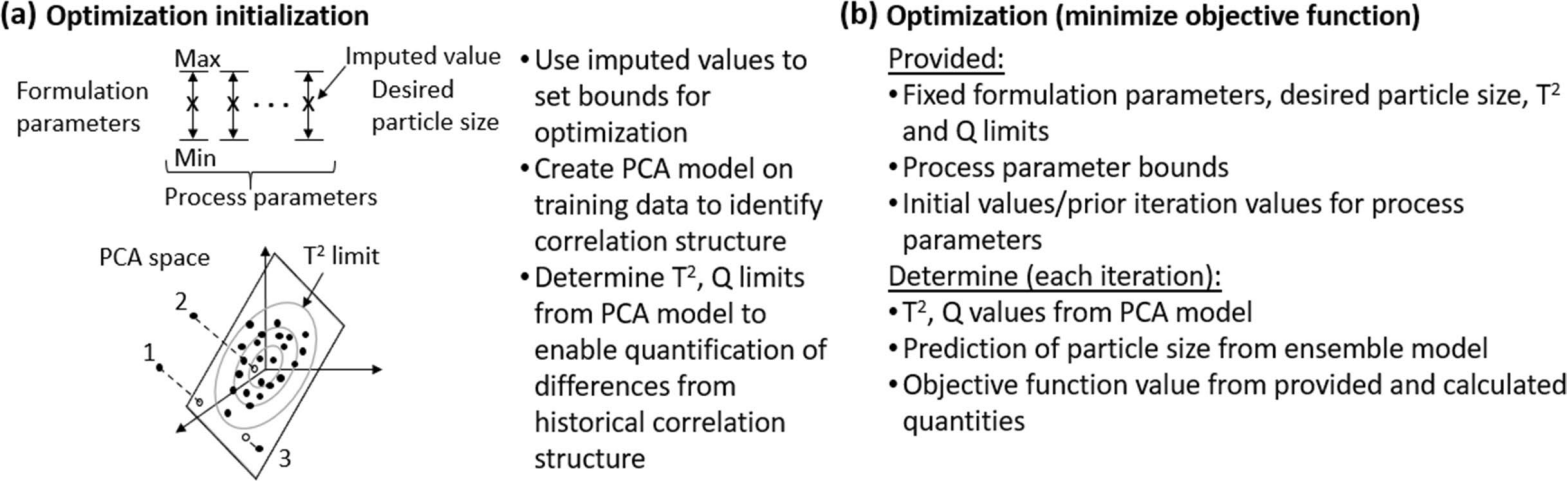
Overview of optimization employed in the second phase of model inversion. (**a**) The optimization is initialized by determining bounds for each of the process parameters from the imputation result. A principal component analysis model is created to determine the correlation structure of the historical data and control limits (T^2^, Q) that define when the correlation structure is violated. Violations of the T^2^ limit correspond to points whose projection onto the hyperplane defined by the principal components are outside of the ellipse defining the T^2^ control limit. Violations of the Q limit occur for points that are sufficiently far away from the hyperplane defined by the principal components. Points 1, 2 and 3 illustrate violations of: both the T^2^ and Q limits, the Q limit and the T^2^ limit, respectively. (**b**) Optimization inputs and process employed to determine process parameters that minimize the objective function.

An optimization strategy is employed in the second phase (Fig. 4) to determine the set of process parameters within the established bounds that best achieve the target particle size, as computed by the particle size prediction model. To ensure that the resulting solutions are physically relevant, the optimization objective function includes terms that quantify how different the proposed solution is from formulation/process parameter patterns present in the training data set. Specifically, process parameters were optimized using a derivative-free optimization strategy (a modified LIPO optimizer, as present in the dlib toolbox) in conjunction with the developed predictive model of dried particle size [35, 36]. For each combination of process parameters evaluated by the optimizer, the process parameters and fixed formulation parameters are supplied to the model and a prediction of the associated dried particle size is returned. As a wide variety of combinations of process parameters within the specified bounds could potentially achieve similar predicted dried particle sizes, the correlation structure present in the original training set is employed to help guide potential solutions. Specifically, principal component analysis (PCA) models are constructed from training data subsets associated with the PSD-1 and PSD-2 scale dryers to define the correlation structure at each scale. For each PCA model, upper control limits are identified for both the Q and Hotelling T^2^ statistics to quantify how well a combination of process and formulation parameters adhere to the correlation structure present in the historical data [37]. Given a fixed formulation and a combination of process parameters supplied by the optimizer, the associated Q and T^2^ values can be computed and compared to these control limits. Values exceeding the established Q or T^2^ control limits indicate that the selected parameter combination breaks the correlation structure, or is an outlier with respect to the associated training data, respectively. Including a penalty in the optimization objective function for combinations that exceed these control limits implicitly incorporates historical knowledge and subject matter expertise to help guide the optimization to more physically relevant solutions.

## Results

### Predictive Model for Dried Particle Size

#### Construction of Training and Testing Data Sets and Data Preprocessing

To cluster data for data splitting purposes, the attributes that generate the best clustering of sprays and APIs were identified using an evolutionary algorithm, similar to that employed for model hyperparameter optimization. Details regarding the evolutionary algorithm for clustering and subsequent data splitting are provided in the Supplemental Material. Primary characteristics of the training and testing data sets established by randomly sampling APIs from these final clusters are presented in Table II.

**Table II:** Training and Testing Data Set Characteristics

Data Set	Number of APIs	Number of PSD-1 sprays	Number of PSD-2 sprays
Training	65	360	119
Testing	23	145	56

In addition to the data set characteristics specific to the scale of the spray dryer (PSD-1 or PSD-2), of the 57 nozzle identifiers present in the collected data, the training and testing data sets contained 44 and 25 unique nozzle identifiers, respectively. Thirteen of the 25 nozzles present in the testing data set, associated with 54 of the 201 sprays, were not contained in the training data set. The nozzle target encoding for final model development was established on the 44 unique nozzles present in the training data set using the TargetEncoding function within the Category Encoders Python package (v2.2.2) with k = 1 and f = 3. These characteristics are highlighted given the importance of the droplet size generated by the atomizer directly impacting final particle size. This ensured that the entire range of nozzle types and sizes were captured across the range of data present and would ideally capture prediction of new cases more accurately.

#### Model Development

Regression models were developed to predict the dried particle size from provided formulation and process parameters. A variety of regression modeling strategies were evaluated, including: partial least squares (PLS), regularized linear regression (ridge, lasso, elastic net), multivariate adaptive regression splines, support vector regression (SVR), kernel ridge regression, random forest, extreme gradient boosted trees (XGBoost) and neural networks (multi-layer perceptron). All modeling strategies, except for XGBoost and neural networks, were created using the scikit-learn framework (v0.23.2) in Python (v3.7) [34]. Neural networks were created using Keras in Tensorflow (v2.1.0), while extreme gradient boosted trees were created using XGBoost (v1.0.2) [38, 39]. Model hyperparameters in each instance were optimized using the training data set and the evolutionary algorithm described in the Supplemental Material. An initial population of 200 hyperparameter sets was established for the evolutionary algorithm; for each iteration of the algorithm, a subset of 40 hyperparameter sets was randomly extracted from the population and the set with the best cross-validation performance was mutated and evaluated. The evolutionary algorithm was run for 800 iterations to minimize an objective function comprised of the leave-one-group-out cross-validation root mean square error (RMSE). Ensembles of individual models were also considered in which the average of the associated predictions from each individual model in the ensemble was employed as the output prediction. No additional training was performed in ensemble creation; all possible ensemble combinations were created and evaluated in terms of their performance.

**Fig. 5:**
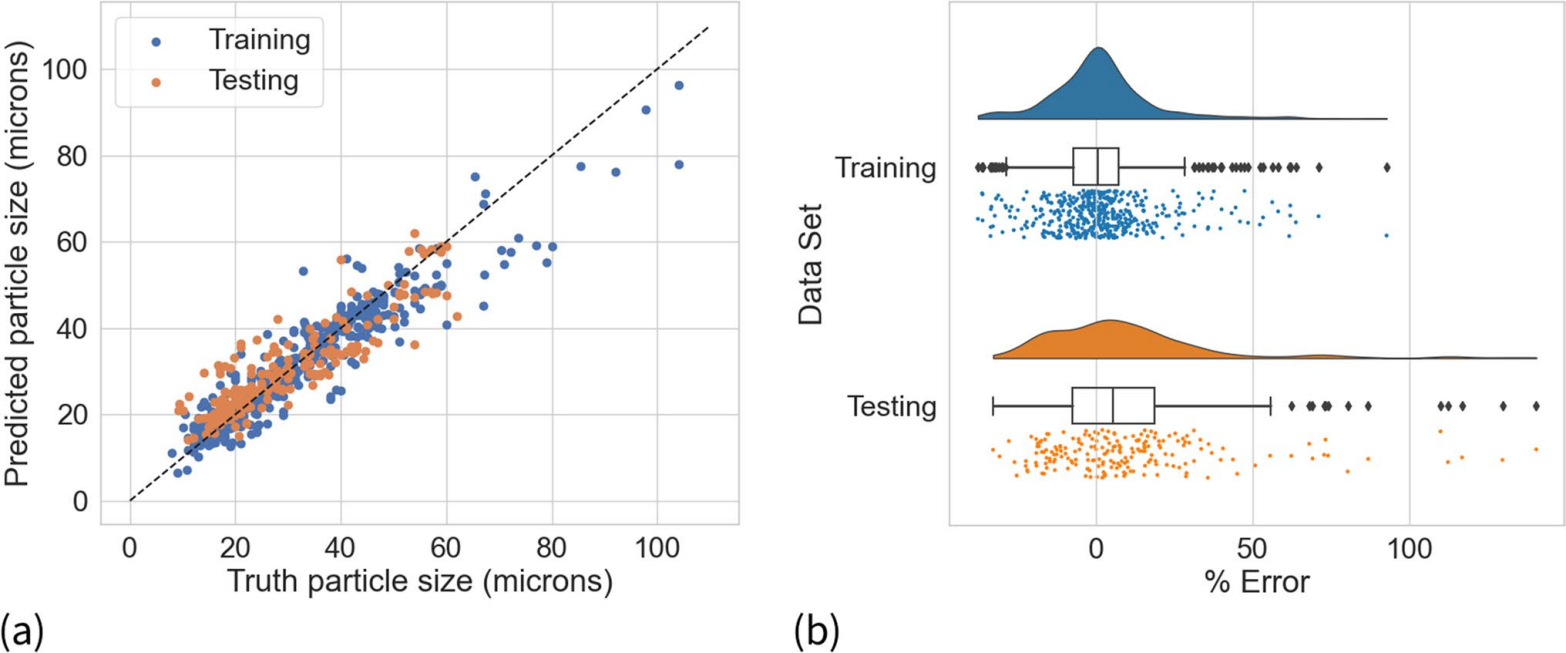
(**a**) Ensemble predictions of median dried particle size as compared to the true values for the training (blue) and testing data sets (orange). (**b**) Raincloud plot [40] illustrating the distribution in the percent error of the model predictions for the training (blue) and testing data sets (orange), as compared to the recorded dried particle sizes.

Modeling results for a selection of the best performing models and their optimized hyperparameter values are presented in Table III. Tree-based methods (random forest, XGBoost) were found to overtrain, yielding comparatively poorer performance on the test data set. Non-parametric models (SVR and kernel ridge, both with a radial basis function kernel) performed well, while linear models and their associated variations trailed slightly behind. The neural network model obtained the best performance of the models considered. An ensemble model comprised of PLS, SVR and the neural network had marginally improved performance on the test set and a smaller difference between training and testing performance than the neural network alone. Ensemble model predictions for the training and testing data sets, as compared to the associated truth values, are illustrated in Fig. 5. The ensemble model generally does a good job of predicting the dried particle size for sprays of previously unseen APIs, with the 25th and 75th percentiles of the prediction percent error of test set sprays identified as −7.7% and 18.6%, respectively. While the largest percent error values evidenced in the test set occurred for some of the smallest recorded particle sizes, the test set also included sprays for similar particle sizes for which the model was more accurate.

**Table III:** Model Performance and Optimized Hyperparameters

Model Type	CV RMSE (microns)	Training RMSE (microns)	Testing RMSE (microns)	Optimized hyperparameters
PLS	9.79	7.69	6.81	Number of components = 8
Ridge	9.93	7.67	6.80	Alpha = 0.78
Lasso	9.80	7.71	7.09	Alpha = 0.027
SVR	10.05	5.39	6.56	Kernel = radial basis function (‘rbf’) C = 79.91 Epsilon = 0.035
Kernel Ridge	10.38	6.59	6.73	Kernel = radial basis function (‘rbf’) Alpha = 0.075 Gamma = 0.075
XGBoost	9.04	4.42	7.23	Number of estimators = 92 Colsample_bytree = 0.79 Gamma = 1.0 Min_child_weight = 7.21 Subsample = 0.58 Max_depth = 3 Eta = 0.1 Alpha = 0 Lambda = 1
Neural Network	5.61	3.72	6.10	Number of neurons per layer = [30, 70] Dropout per layer = [0.05, 0.05]
Ensemble: SVR, NN, PLS	Not applicable	5.09	6.09	Not applicable

The resulting RMSEs were 5.09 and 6.09 for the training and test sets, respectively. As detailed in the Supplemental Material, the corresponding ensemble model built with nozzle orifice diameter and without target encoding yielded RMSEs of 6.72 and 6.65 for the training and test sets, respectively. The difference in performance (encoding *versus* simple orifice diameter) suggests that while target encoding is not solely responsible for the model performance achieved, it does improve performance by encapsulating information about nozzle/fluid interactions beyond those that could be determined through nozzle orifice diameter alone. Using both target encoding and nozzle orifice diameter in model development did not yield performance improvements beyond that achieved with target encoding alone. This ensemble model can be most directly compared with results reported from a statistical DoE study performed during telaprevir design space development. A design space was constructed from 162 total experiments using various factorial designs and a particle size regression was created with a RMSE of 7.3 [20]. The telaprevir studies were performed with a single API and formulation with commercial scale spray dryers (e.g. FSD 12.5 and PSD-4). This highlights both the associated challenges with creating a statistical particle size model even for a single formulation and also the relative success of the broadly applicable developed model.

### Determining Process Parameters to Produce a Desired Dried Particle Size

The ensemble dried particle size prediction model was employed in the model inversion strategy described previously to determine ‘first to try’ process parameters for a prescribed formulation and desired dried particle size. The imputation strategy (Fig. 3) was used to determine the bounds for each of the process parameters for the subsequent optimization step. For each spray, the proposed formulation, spray dryer scale and desired median dried particle size were specified. These values were placed into an array in the same order as they were present in the training data set and the missing process parameters filled with not a number (‘NaN’) values. After appending the array onto the subset of the original training set associated with the same spray dryer scale, the data set was passed to the imputation function. The iterative repeated imputation was performed using the IterativeImputer function in scikit-learn with a Bayesian Ridge Regression model [33, 34]. Bounds on the process parameters during imputation were set to the minimum and maximum values evidenced in the training data set for the associated spray dryer type, except for the nozzle encoding values. Minimum and maximum bounds on the nozzle target encoding values were set to 75% and 125% of the target dried particle size. The iterative imputation was performed for 20 iterations in a random fashion with the initial values of each missing process parameter set to the mean values of the data provided to the imputation function.

While the imputation determined estimates of the process parameters from the data present in the training data set, it did not ensure that these estimates produce the target dried particle size or adhered to the correlation structure present in historical data. The process parameters identified via imputation were thus employed in establishing the bounds on the process parameters for the subsequent optimization of the predicted dried particle size. Bounds on the inlet temperature, outlet temperature, drying gas flowrate and solution to drying gas flowrate ratio were set at 90% and 110% of the identified values from the imputation. Bounds on the atomization pressure and nozzle encoding were set at 50%/150% and 75%/125% of the identified values from the imputation, respectively. PCA models were constructed on the subsets of the training data associated with the PSD-1 and PSD-2 scale spray dryers to enforce correlation structure in the optimization solution. In each case, the number of components employed in final PCA model development was set to the average of the number of components suggested from six different strategies, including: eigenvalue test, parallel analysis, random average under parallel analysis, random average under permutation and two randomization methods based upon eigenvalues [41]. The Q and T^2^ control limits (*Q*_*UCL*_, ${T}_{UCL}^2$) were calculated for each model according to previously established relationships, with a confidence limit of 99.73% [37]. The objective function for the optimization was constructed as:

$$J=\left|y-\hat{y}\right|+100H\left(Q-{Q}_{UCL}\right)\left(\frac{Q}{Q_{UCL}}-1\right)+100H\left({T}^2-{T}_{UCL}^2\right)\left(\frac{T^2}{T_{UCL}^2}-1\right)$$

where y is the desired median dried particle size, $\hat{y}$ is the predicted dried particle size, H is the Heaviside step function and Q and T^2^ are the values calculated for the instance from the PCA model associated with the indicated spray dryer scale. As indicated in the objective function, the penalties based upon violations of the correlation structure, as measured by the Q and T^2^ statistics, begin at zero at the associated control limit due to the Heaviside step function and increase linearly with increases beyond the control limit. Optimization of the objective function was conducted within the prescribed bounds using the find_global_min function of the dlib library using 200 iterations and the epsilon solver parameter set to 10% of the desired particle size [35]. Upon completion, an optimization was considered successful if the predicted particle size was within 10% of the desired size and neither control limit was exceeded for the Q and T^2^ statistics.

**Fig. 6:**
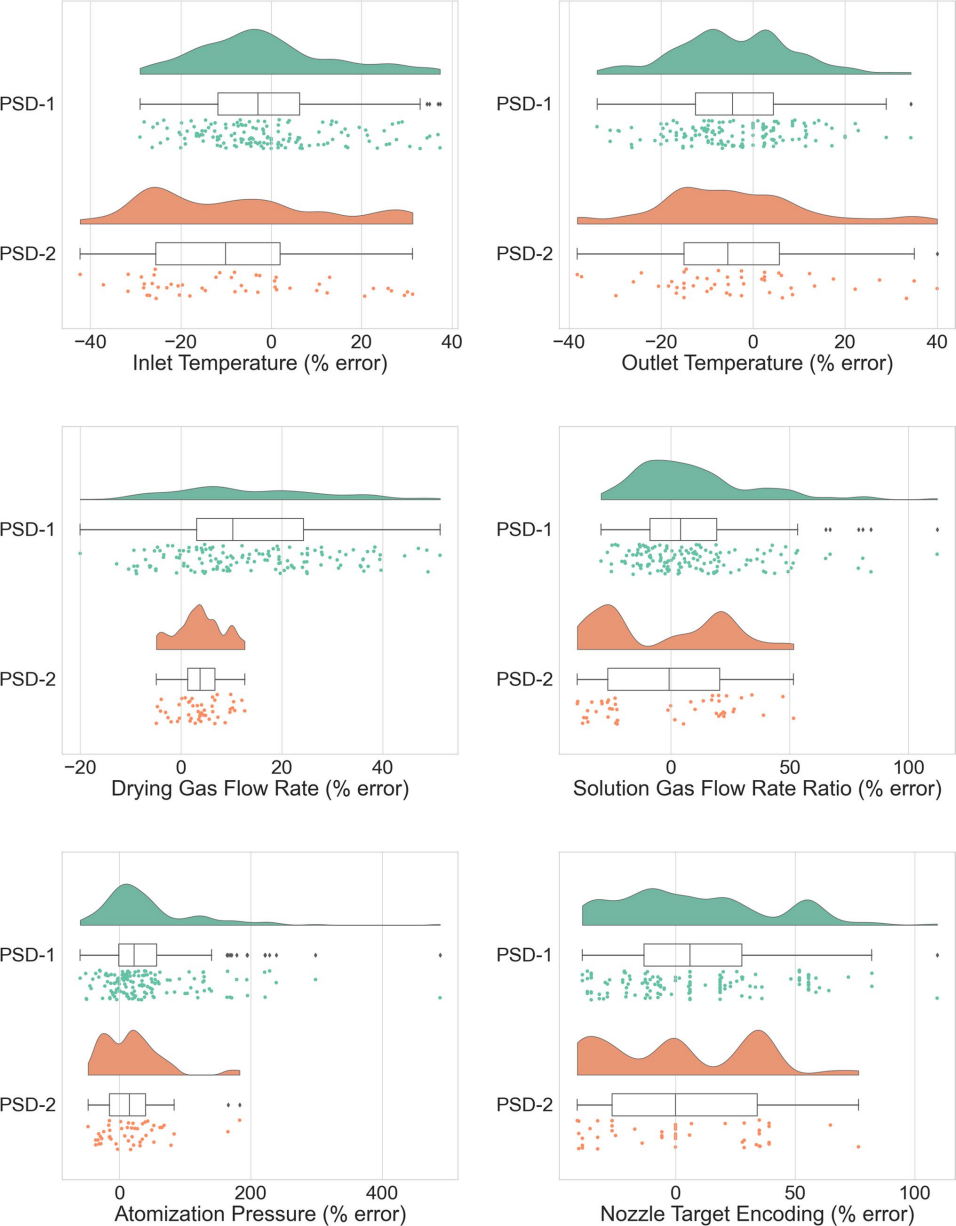
Raincloud plots of the percent error in the estimated process parameters for sprays present in the test data set, split by spray dryer size, as computed by the model inversion strategy.

This optimization strategy was conducted to obtain estimates of the process parameters employed for all of the sprays in the test data set. The percent error in the estimated process parameters was calculated for each parameter, as illustrated in the raincloud plots (Fig. 6) and summarized in Table IV [40]. The optimization strategy successfully produced results for 191 of the 201 instances of the test data set. Inversion failures occurred primarily due to violations of correlation structure and were associated with instances where the recorded atomization pressure was on the low end of values present in the training data set. Failures due to correlation structure violations were not entirely surprising, as outliers with respect to the correlation structure existed within the PCA models constructed on the training data set. As illustrated in both Fig. 6 and Table IV, this strategy had comparatively better success in estimation of the temperatures, flow rates and nozzle target encoding than it did for the atomization pressure. Errors in inversion estimates are perhaps unsurprising considering that this strategy attempts to employ a many to one model (formulation/process parameters to particle size) in an inverse fashion (one particle size yielding an estimate of several process parameters for a provided formulation). As noted previously, inversion failures were noted due to low atomization pressure which creates larger particle sizes. The median particle size in this study ranged an order of magnitude; some of the larger particle size data would be considered outside of a typically targeted value and was studied experimentally well below the lower threshold of atomization pressure where the spray plume is fully developed and operates in a robust fashion. These results could have also been skewed due to low process yield or particle agglomerates due to the longer drying times required for that droplet size. Establishing a lower bound of atomization pressure based on atomization quality would be a practical way to further refine the accuracy of the inversion model and also provide scientists with a way to further refine process conditions during development and subsequent scale-up.

**Table IV:** Process Parameter Estimation Error Summary

Parameter	Spray Dryer	Median % error	% error (25th percentile)	% error (75th percentile)
Inlet Temperature	PSD-1	−3.0	−11.7	6.4
	PSD-2	−10.1	−25.5	1.9
Outlet temperature	PSD-1	−4.4	−12.5	4.4
	PSD-2	−5.5	−15.0	5.8
Drying gas flow rate	PSD-1	10.3	3.1	24.3
	PSD-2	3.8	1.4	6.7
Solution gas flow rate ratio	PSD-1	4.1	−8.9	19.3
	PSD-2	−0.6	−26.5	20.5
Atomization pressure	PSD-1	22.2	−1.2	56.6
	PSD-2	15.5	−15.2	39.6
Nozzle target encoding	PSD-1	6.1	−13.3	27.9
	PSD-2	0.0	−26.5	34.1

The performance of the model inversion strategy was also examined for use in scale-up between PSD-1 and PSD-2 dryers. Two APIs present in the test set were retained for evaluating model inversion performance as they had sprays at both scales with consistent formulations and particle sizes. Scale-up presents a unique case in model inversion, as it is often performed targeting the same solution to drying gas flow rate ratio and the nozzle for the PSD-1 spray has already been identified from initial development work establishing a particle size target. These known values were employed in establishing the lower and upper bounds in both the initial imputation and subsequent optimization for the process parameters associated with the PSD-2 spray. While this examination was limited to a very small sample size, the model inversion showed promise for identifying initial process parameters for scale-up in these instances. As illustrated in the results of Table V, the model inversion process yielded relatively small percent errors in estimates of process parameters from those actually employed in the associated PSD-2 sprays.

**Table V:** Identifying PSD-2 Operating Conditions from PSD-1 Conditions

Inlet temperature (% error)	Outlet temperature (% error)	Drying gas flow rate (% error)	Solution to drying gas flow rate ratio (% error)	Atomization pressure (% error)	Nozzle target encoding (% error)	T^2^/(T^2^ control limit)	Q/(Q control limit)	Desired particle size, inversion particle size (microns)
5.7	−4.0	−7.1	−8.5	−38.0	−26.5	0.76	0.78	21.0, 21.6
−7.4	−18.4	8.5	6.4	−5.5	−11.6	0.68	0.40	20.0, 20.0

## Discussion

SDD particle size has a nonlinear dependence on API, formulation and process parameters. API and excipient properties such as melting point, glass transition temperature, viscosity and solvent solubility can all potentially impact particle morphology and resulting particle size. Even for a given API and formulation, numerous experiments are often required to determine process parameters that achieve the desired particle size for a specific product [20, 21]. In this study, we show that historical formulation and process parameters across a range of APIs can be leveraged by machine learning techniques to predict median dried particle size.

### Deriving Insight from the Machine Learning Model

While the machine learning model does not directly provide a mechanistic explanation of particle size as a function of the input parameters, it can be used to identify important input parameters and their impact on particle size, as shown by the associated SHAP values. This provides insights to pursue more mechanistic studies that may be important for a specific API and formulation, or to further examine in the context of what has already been described in the literature.

**Fig. 7:**
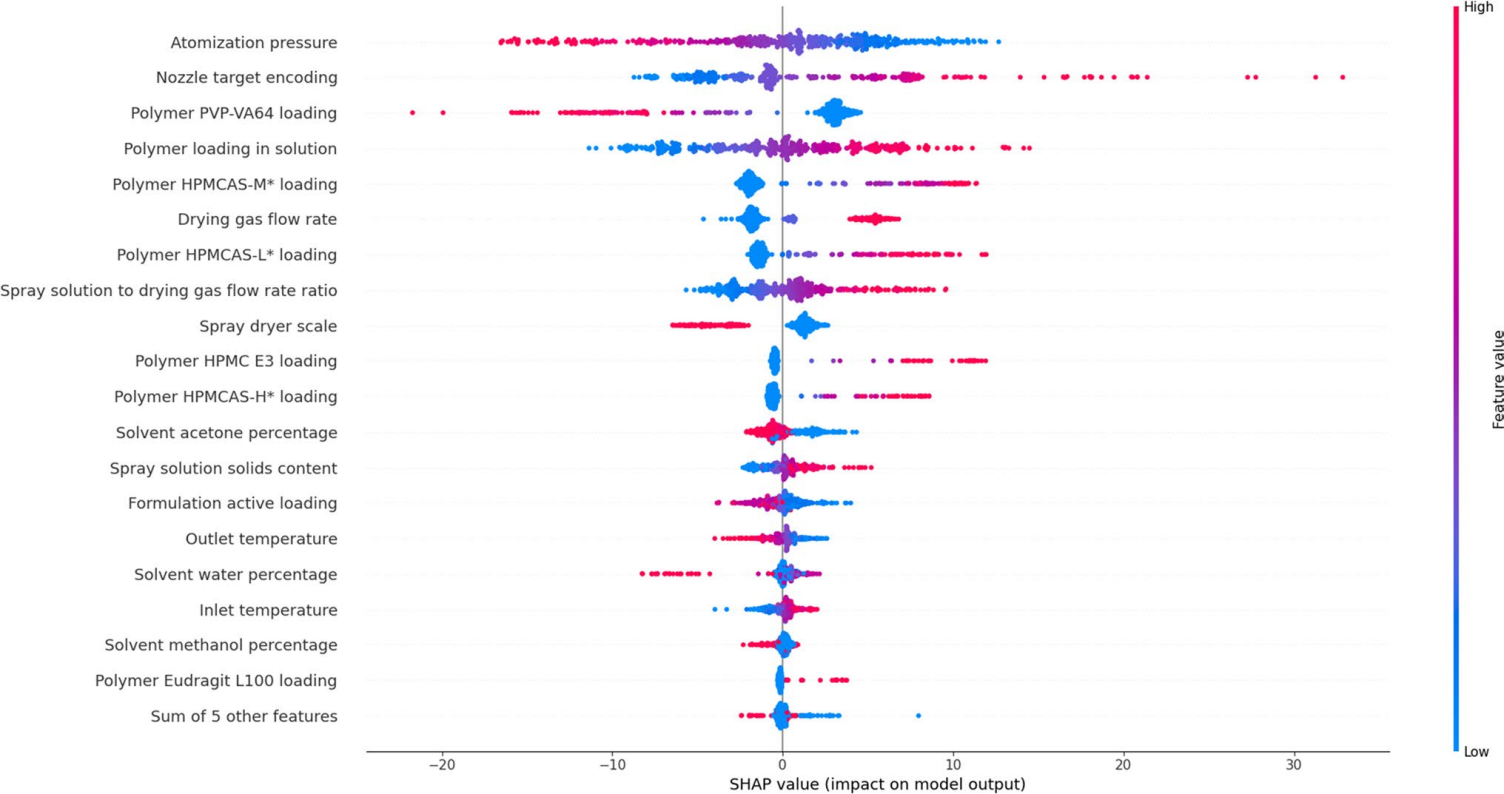
Beeswarm plot illustrating the magnitude and direction of the contribution (SHAP value) of attributes to the ensemble model prediction of dried particle size for the training data set. Each dot represents an instance of the training data set. Color indicates the relative value of the attribute in comparison to the overall range evidenced for the attribute in the training data set (blue = smaller, red = larger). Distance along the x-axis indicates the positive (or negative) contribution towards the ensemble model prediction for the attribute and instance (>0, <0 increases/decreases the particle size prediction).

SHAP values were computed for each model of the ensemble using the SHAP package (v0.39), with ensemble SHAP values computed as the mean of the SHAP values from each model within the ensemble. Attribute contributions to ensemble model predictions are illustrated for the training data set via SHAP values in the beeswarm plot (Fig. 7). Attributes are rank-ordered in terms of the absolute magnitude of their contributions to the output predictions. The beeswarm plot facilitates interpretation of attribute effects on ensemble model predictions in a global sense and the relationships identified via SHAP values (Fig. 7) appear to reflect relationships identified between both formulation parameters and/or process parameters and droplet/particle size in prior research. For example, increases in atomization pressure have been demonstrated experimentally to yield smaller particle sizes due to higher atomization energy, which are represented in Fig. 7 by negative contributions to the particle size prediction as atomization pressure increases [28]. Increases to the solution flow rate have been demonstrated to increase particle size due to an increased amount of liquid to break up for constant atomization pressure [13, 14]. In the model, this is reflected by increases in the solution to drying gas flow rate ratio yielding positive SHAP contributions to the model predicted particle size. The trends in contributions to the predicted particle size via the SHAP values (Fig. 7) and particle sizes demonstrated experimentally are also borne out for the solution solids content and organic solvents, which are both variables that contribute to solution viscosity [13, 14, 26]. Larger droplets and subsequent particles are formed from higher viscosity solutions due to the increased energy required to break-up the spray into droplets. An additional effect from solution solids content is due to the shrinking of the droplet to a solid particle that is dependent upon solids concentration when applying a shrinking sphere to model particle formation where particle size is proportional to (solids concentration)^1/3. Increased polymer loading in the formulation increasing particle size likely relates to both particle density changes and differences in spray solution viscosity. Decreasing the polymer loading in the SDD (i.e. increasing API loading) would trend to denser particles due to differences in solidification behavior between a polymer molecule and an API molecule, therefore, leading to a smaller observed particle size. Spray solution viscosity effects are driven by the solution solids content where API solubility typically limits solids concentration In the spray solution. Increasing polymer loading would thus tend to increase the overall spray solution viscosity given the increased concentration of polymer at a constant API concentration.

While increases in drying gas flow rate appear to yield increases in predicted particle sizes (Fig. 7), isolating the instances associated with positive SHAP values for the drying gas flow rate attribute revealed that these instances included all of the PSD-2 scale instances of the training data set and a handful of PSD-1 instances, all of which demonstrated elevated drying gas flow rates in comparison to the remaining PSD-1 instances. As such, it appears that the SHAP values associated with the drying gas flow rate are effectively capturing differences in scale. This outcome is intuitive because the PSD-2 scale spray dryer operates with ~5× higher flowrates than the PSD-1 dryer. The higher flowrates also require larger nozzles, both of which would tend to lead towards larger particle sizes. Additionally, during scale-up larger particle sizes are achievable and can be targeted to improve SDD flowability or compressibility that are important quality attributes for dosage form manufacture. Drying gas flowrate is also captured in the solution to gas flow ratio attribute, where decreases in the SHAP values for lower solution to gas flow rate ratio values are capturing the smaller particle sizes that one would expect to be evidenced for decreased liquid feed rate at a given scale of dryer [14].

**Fig. 8:**
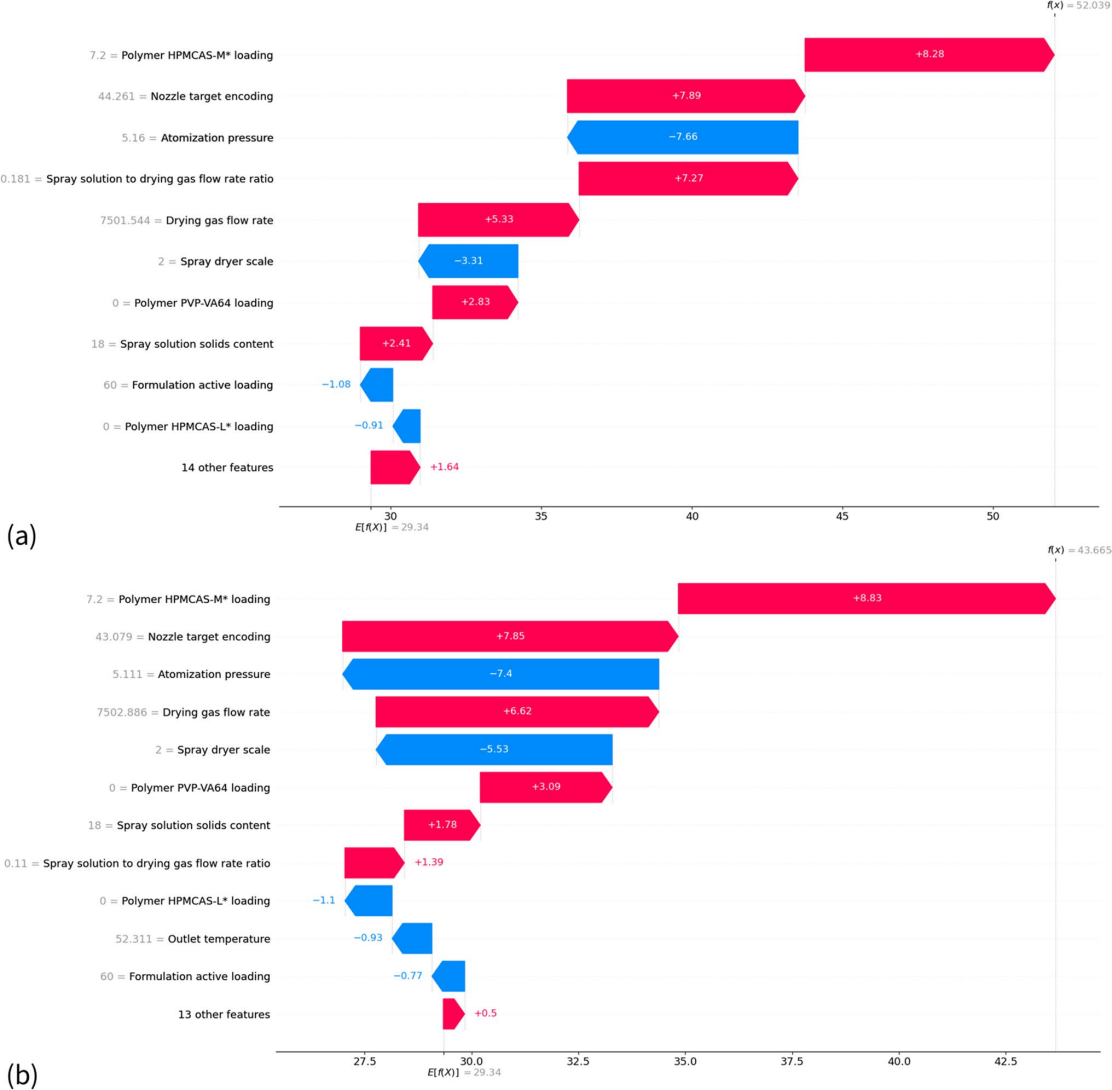
Waterfall plot of attribute SHAP value contributions to the overall ensemble model prediction for a two instances of the training data set associated with the same API and formulation parameters. For subplots (**a**) and (**b**), the ensemble model prediction and recorded particle sizes were, in microns: (52.04, 54.02), (43.66, 44.55). In each plot, blue and red bars indicate the attribute contribution to the model prediction (blue = negative, red = positive) with the associated magnitude as indicated. Numbers to the left of each attribute indicate the attribute values for the provided instance. The sum of all attribute contributions, combined with the mean overall model prediction (E(f(X)), results in the ensemble model prediction as denoted by f(x) in the top right portion of each plot.

Locally, a model prediction for particle size can be explained using waterfall plots (Fig. 8). The waterfall plot provides the contribution of each attribute to the model prediction ordered in decreasing magnitude, which may not have the same order as shown for the overall results of the beeswarm plot (Fig. 7). Indeed, the waterfall plots (Fig. 8) illustrate that polymer loading was actually the most important parameter in predicting particle size for these two sprays, but not for the global fits. The waterfall plot could be used in conjunction with the beeswarm plot (Fig. 7) to manually refine the choice of input process parameters for a prescribed formulation to achieve a desired model-predicted particle size. For example, the sprays of Fig. 8 illustrate model predictions for the same API with constant formulation parameters but differences in several process parameters. Maintaining the atomization pressure and drying gas flow rate at approximately constant values between the two sprays, the SHAP value contributions from the beeswarm plot (Fig. 7) suggest that a decreased particle size could be obtained by: choosing a nozzle with a smaller encoding value (i.e. smaller nozzle orifice) or decreasing the spray solution to drying gas flow ratio by decreasing the liquid flowrate as drying gas flowrate is typically held constant at a given scale. It is also observed that decreasing particle size could also be accomplished by increasing the outlet temperature and/or decreasing the inlet temperature; however, these would be considered secondary effects that co-vary with decreases in solution feed rate which would lead to a smaller droplet size but changes to temperature would likely have a small or negligible effect on final SDD particle size based on previous studies [25]. Indeed, the second spray of Fig. 8 was conducted with the following changes: a large decrease in the spray solution to drying gas flow ratio (0.181 to 0.11), a large increase in the outlet temperature (34.0 to 52.3 degrees Celsius), a nozzle with a slightly smaller target encoding value (44.261 to 43.079) and a small reduction in the inlet temperature (120.2 to 112.4 degrees Celsius). These changes resulted in a 8.38 μm decrease in the model prediction of particle size as compared to a 9.47 μm decrease in the recorded particle size. As expected from the process parameter changes and the SHAP contribution trends (Fig. 7) for these attributes, the largest contributions to the decrease in model prediction particle size were due to the contributions from the spray solution to drying gas flow ratio (5.88 μm, from 7.27 to 1.39) and the outlet temperature (1.53 μm, from 0.6 to −0.93). These results (Fig. 8) illustrate how scientists could employ a predictive model in combination with SHAP contributions to refine process parameter values in advance of experimental evaluation. Furthermore, an iterative in silico design space could be developed, which is particularly important during scale-up.

### Implications for Model-Assisted Development

That the ensemble model, via the calculated SHAP values, was able to capture previously identified relationships between process parameters and particle size that have underlying mechanistic explanations suggests that SHAP values could be used for hypothesis generation for future investigations. These results also imply that further quantification of nozzle characteristics could be beneficial to the understanding of particle size, due to the comparatively large contributions of nozzle encoding to the predicted particle size. A better characterization of the set of interacting factors, such as atomizer internal geometry and shearing/inertial forces, that are potentially being encapsulated in the nozzle encoding attribute could drive a better understanding of the relationship between nozzle properties, droplet size and subsequent particle size.

Explanation of model predictions through the waterfall plot provides scientists an in-silico means to understand how changes in operating parameters could impact the particle size before running an experiment. Additionally, the model inversion optimization could help scientists identify an initial set of process parameters to refine that implicitly incorporate a holistic compilation of historical knowledge. While the inversions conducted in this proof of concept analysis were performed without any subject matter expertise, it is possible improved solutions could be obtained by scientists providing more appropriate search ranges for the process parameters in the inversion optimization. Additionally, the model provides a framework for defining initial targets for particle size, key attributes for controlling particle size and a means to initiate process development using a QbD approach. Finally, using a predictive particle size model during scale-up can significantly decrease experimental time and API consumption while providing a high level of confidence on the proposed process.

The results of this study demonstrate that machine learning can be used to derive value from measurements routinely collected during spray drying, provided that they are collected with enough variation to establish patterns that span the operating space expected in routine operation. Although the models developed here were aimed at predicting median particle size, other attributes can also be predicted using these techniques, including other particle size distribution metrics (e.g. D10, D90 or span), or other key particle features, such as bulk density or flow properties. These features could likely be predicted from the same input data used in the current study because it contains both formulation and process attributes that are important to defining these parameters.

## Conclusions

Understanding the relationship between formulation parameters, process parameters and dried particle size is an important part of the process development workflow for bioavailability-limited drugs. As drug development timelines become increasingly compressed, it is important to both minimize the amount of experimentation while also maximizing confidence in a right-first-time approach. In this study, an ensemble machine learning model was created to predict dried particle size from formulation and process parameters. It is believed that this is the first model that is applicable across a diverse range of APIs, formulations, scales and process conditions to predict median dried particle size. The constructed model, in conjunction with Shapley additive explanations, enables better understanding of how variations in the process parameters for a given API and formulation will affect the dried particle size. The developed optimization strategy leverages both the predictive model and the historical knowledge implicitly included in the training data set to provide scientists with initial estimates of the experimental process parameters that will yield a specific particle size. Combined with their subject matter expertise, scientists could leverage these results to reduce the amount of experimental effort required to identify acceptable process parameters associated with a specified particle size during process development. It also allows identification of key formulation or process attributes that could be studied in more detail for a specific API. Finally, the approach could be applied to development of an in silico design space that can be confirmed with significantly reduced effort compared with traditional DOE using a QbD framework.

## Supplementary Information

ESM 1 (PDF 280 kb)
