# Supplementary material for: Predicting Spray Dried Dispersion Particle Size Via Machine Learning Regression Methods
Source: Pharm Res. 2022 Aug 19;39(12):3223–39. doi: 10.1007/s11095-022-03370-3 (PMC9780133; doi:10.1007/s11095-022-03370-3)
Supplement: Supplementary file 1 — (PDF 280 kb) [file 11095_2022_3370_MOESM1_ESM.pdf]

Target encoding

In the target encoding strategy, each nozzle identifier is replaced with the weighted average of the particle size values associated with the nozzle and the overall average particle size, as given by the formula:

$$Encoding_i = \lambda(n_i)(mean\ size)_i + \big(1 - \lambda(n_i)\big)(overall\ mean\ size)$$

$$\lambda(n_i) = \frac{1}{1 + e^{-\frac{n_i - k}{f}}}$$

where n_i indicates the number of instances of nozzle i in the dataset, (mean size)$_i$ is the corresponding mean particle size associated with the instances of nozzle i, k is a sample size parameter that influences how much trust is placed in the estimates based upon the sprays associated with nozzle i and f is a smoothing parameter that controls the rate of change between trusting the mean particle size associated with the nozzle i and the overall mean particle size obtained across all experiments (1). In this study, k and f were set to values of 1 and 3, respectively. As n_i decreases, the weighting on the overall mean particle size evidenced increases in determining the encoded value. While the encoded value is primarily determined by the particle sizes produced for a specific nozzle as n_i increases, the variety of APIs, formulation and process parameters also increases leading to a wider expected range of particle sizes, thereby decreasing the chance of overfitting.

Clustering strategy for splitting data into training and testing data sets

The approach employed for splitting the provided data into training and testing sets is summarized in Fig. S1. Initially, all sprays were clustered using an evolutionary cluster feature selection algorithm with a density-based clustering technique (HDBSCAN), with the validity index representing clustering quality (2, 3). This strategy identified the formulation/process parameters to use in clustering and minimum cluster size that produced the tightest clusters of sprays with the greatest distances between them. The resulting spray clusters could contain sprays associated with multiple APIs and sprays for a particular API could be spread across clusters. As a result, these spray clusters could not be directly used to establish the training and testing sets, as randomly sampling from them could lead to sprays associated with an API being added to both the training and testing sets. However, APIs could be clustered under the assumption that APIs were similar if their associated spray attributes split in a similar manner across the spray clusters. For each API, the fraction of sprays present in each spray cluster were identified and formulated as a vector. Cosine distance was computed on these vectors across all APIs as a similarity metric. HDBSCAN was employed again on these distance vectors to determine clustering of APIs that had a similar split in sprays across the previously identified clusters. Random selection of APIs, and associated sprays, from these API clusters was used to create the training and testing sets. In this fashion, all sprays associated with an API were included in either the training or testing data set, but not split across both.

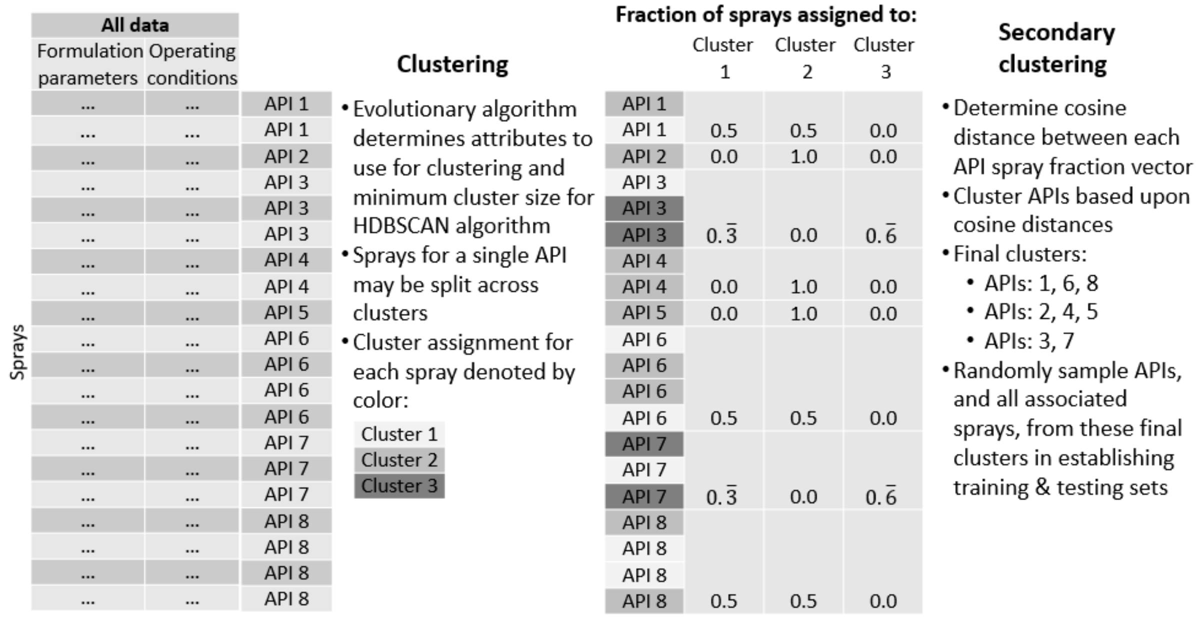

Fig. S1 Illustrative example of strategy used in assigning sprays associated with APIs to the training or testing data sets. An evolutionary cluster feature selection algorithm applied to the entire data set results in clusters of sprays, where sprays associated with a given API may be split across clusters. APIs are clustered based upon similarity in how sprays for each API are split across the spray clusters. Training and testing data sets are established by randomly sampling APIs, and all of their associated sprays, from the final API clustering.

Evolutionary algorithm for model hyperparameter optimization

Model hyperparameter optimization was performed using an evolutionary algorithm to optimize the leave one group out cross-validation performance on the training data set (Fig. S2). Specifically, a regularized tournament selection algorithm was employed in model hyperparameter optimization (4). In this strategy, an initial population of different model hyperparameter choices was constructed, where the quality of those choices was quantified by the associated leave-one-group-out cross-validation performance. For each member of the population, the model hyperparameter values were randomly selected from uniform distributions between prescribed bounds and the leave-one-group-out cross-validation performance was computed. After constructing this initial population, the evolutionary algorithm was employed to identify how these hyperparameter sets could be changed to achieve better cross-validation performance. In each iteration, a subset of the population of was randomly extracted and the model hyperparameter set with the best cross-validation performance within the subset was selected for mutation. In the mutation step, one of the model hyperparameters from the selected set was changed in a random fashion from the provided value. The combination of random subset selection and mutation balances exploration of the search space with exploitation of high performing hyperparameter sets. Using a subset of the population in the selection step enabled weaker hyperparameter sets in the population to become stronger through mutation, as the best overall hyperparameter set may not have been present in the subset. After retraining the model with this mutated hyperparameter set, the new set of model hyperparameters and its associated cross-validation performance was added to the population and the oldest set of model hyperparameters in the population was removed. After the prescribed number of evolutionary iterations, the set of model hyperparameters with the best cross-validation performance was identified from the population and employed in final model training on the entire training data set.

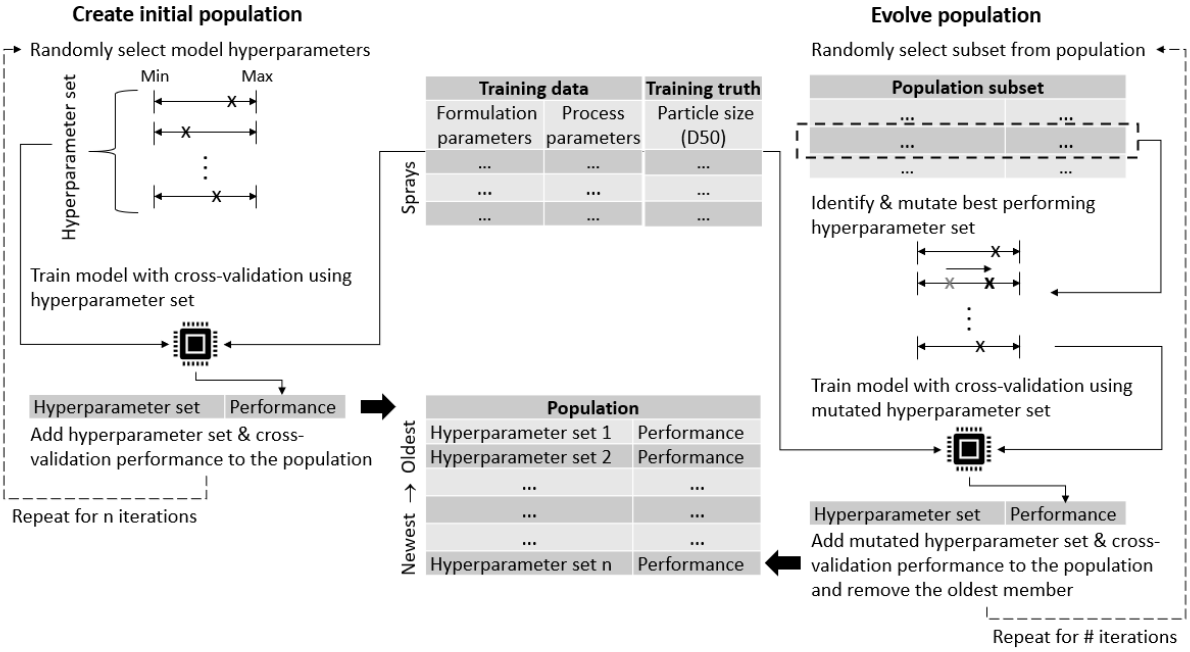

Fig. S2 Overview of the evolutionary algorithm (regularized tournament selection) used in model hyperparameter optimization. An initial population of randomly selected modeling hyperparameter sets and their associated cross-validation performance is evolved by randomly selecting a subset of the population, mutating the hyperparameters associated with the best individual, evaluating the cross-validation performance and updating the population.

Evolutionary algorithm for splitting data into training and testing sets

Initial data splitting into the training and testing data sets was performed using a regularized tournament selection algorithm, similar to that employed for model hyperparameter optimization. In this instance, the initial population of 200 combinations of attributes was constructed by randomly sampling between 3 and 10 attributes for clustering. The evolutionary algorithm was conducted for 600 iterations. At each iteration, a subset of 40 individuals from the population of 200 was extracted and the best performing member of the population, as measured by the validity index of the clustering, was retained for mutation. The attributes of the best performing member were mutated by either adding a new attribute or removing a current attribute, with equal probability. The clustering algorithm present in the HDBSCAN software package (v0.8.26) was run multiple times for a minimum cluster size between 5 and 30 instances (5). The minimum cluster size with the best associated validity index was retained. The mutated instance was added to the population, the oldest member removed and the process repeated for the prescribed number of iterations. The best clustering identified via this strategy was achieved using three attributes: spray dryer inlet temperature, the spray dryer type and whether or not the set of solvents employed included water. This set of attributes, with a minimum cluster size of 14 sprays in the HDBSCAN algorithm, produced 4 clusters with a validity index of 0.95.

As described in the clustering strategy section, for each API a vector was constructed indicating the fraction of the sprays associated with the API that were present in each cluster. Executing the HDBSCAN algorithm again on the cosine distances between these vectors, with a minimum cluster size of 2, yielded 4 clusters. APIs, and their associated

sprays, were randomly extracted from each of these clusters to establish the testing data set.

Model development with nozzle orifice diameter instead of target encoding

The model development process was repeated using orifice diameter as the nozzle-related attribute in place of target encoding. All subsequent modeling activities, including cross-validation and model hyperparameter optimization, were performed in the same fashion. The modeling results and optimized hyperparameter values for models with nozzle orifice diameter are presented in Table SI. As illustrated in Table SI, the performance ranking of the presented modeling strategies was similar to that obtained in the model development process with target encoding. However, the overall RMSE obtained both by individual models and the ensemble exceeded that obtained by using target encoding.

Table SI Model performance and optimized hyperparameters with nozzle orifice diameter used in model development instead of target encoding

Model Type	CV RMSE (microns)	Training RMSE (microns)	Testing RMSE (microns)	Optimized hyperparameters
PLS	10.92	9.39	8.01	Number of components = 7
Ridge	10.73	9.62	7.77	Alpha = 2.656
Lasso	10.61	9.76	8.42	Alpha = 0.109
SVR	10.52	8.40	7.12	Kernel = radial basis function ('rbf')<br>C= 13.95<br>Epsilon= 0.01
Kernel Ridge	11.03	8.91	7.60	Kernel = radial basis function ('rbf')<br>Alpha = 0.316<br>Gamma = 0.075
XGBoost	9.18	3.52	9.25	Number of estimators = 147<br>Colsample_bytree= 0.53<br>Gamma= 0<br>Min_child_weight= 11.20<br>Subsample=0.51<br>Max_depth=4<br>Eta=0.14<br>Alpha=0<br>Lambda=1
Neural Network	4.85	5.15	7.09	Number of neurons per layer = [40, 60, 70, 10]<br>Dropout per layer = [0.0, 0.1, 0.1, 0.0]
Ensemble: SVR, NN, PLS	Not applicable	6.72	6.65	Not applicable

References

1. Micci-Barrreca D. A preprocessing scheme for high-cardinality categorical attributes in classification and prediction problems. ACM SIGKDD Explorations. 2001;3(1):27-32.

2. Campello RJGB, Moulavi D, Sander J. Density-Based Clustering Based on Hierarchical Density Estimates. In: Pei J, Tseng VS, Cao L, Motoda H, Xu G, editors.Advances in Knowledge Discovery and Data Mining PAKDD 2013. Gold Coast, Australia: Springer Berlin Heidelberg; 2013. p. 160-172.
3. Moulavi D, Jaskowiak PA, Campello RJGB, Zimek A, Sander J. Density-based clustering validation. In: Zaki M, Obradovic Z, Tan PN, Banerjee A, Kamath C, Parthasarathy S, editors.Proceedings of the 2014 SIAM International Conference on Data Mining (SDM). Philadelphia. ; 2014. p. 839-847.
4. Real E, Aggarwal A, Huang Y, Le Q. Regularized evolution for image classifier architecture search. In.Proceedings of the AAAI Conference on Artificial Intelligence. Honolulu, Hawaii, USA: AAAI Press; 2018. p. 4780-4789.
5. McInnes L, Healy J, Astels S. HDBSCAN: hierarchical density based clustering. Journal of Open Source Software. 2017;2(11).
